# International Nurse Migration Experience of the First Two Years: A Mixed Methods Study

**DOI:** 10.1111/jan.16543

**Published:** 2024-10-23

**Authors:** Charlene Pressley, Dillon Newton, Linda Sanderson, Bibha Simkhada, John Stephenson, Precious Adade Duodu, Warren Gillibrand, Manju Pallam, Joanne Garside

**Affiliations:** ^1^ The University of Huddersfield Huddersfield UK; ^2^ Manchester Metropolitan University Manchester UK

**Keywords:** cost of living, foreign nurses, housing, international nurse, migrant nurses, National Health Service, overseas nurses, retention

## Abstract

**Aim:**

The aim of this study was to explore experiences of internationally educated nurses' first 2 years working and living in England in an age of contemporary migration.

**Design:**

Exploratory mixed method design.

**Methods:**

An online survey collected responses from August 2022 to October 2022. A mixed‐methods approach was applied to gain a breadth of understanding through quantitative outcomes integrated with depth of qualitative analysis.

**Results:**

Data findings from 773 international nurses identified the recognition of prior professional skills and experiences, induction processes, career development systems and the role of line managers are integral to professional integration and could be improved. Communication barriers were frequently transient and time limited, and participants often had incomplete insight of human resource policies. Personal factors affecting life outside of work revealed compromise and sacrifice with accommodation choices, and participants were often not satisfied with their economic status, housing, cost of living in England, and social support. Mental well‐being scores varied by country of origin and participants happier with decision to migrate to England had significantly higher mental well‐being scores.

**Conclusion:**

International nurses have divergent professional and personal motivations for migration unique to individual circumstances. Recognition for transferable skills and experience, receipt of a comprehensive and warm welcome from organisations, safe and well‐maintained suitable accommodation and living conveniently located to shops, work and transportation, improve experience. International nurses must have sufficient financial gains from salaries and opportunity to unite with children and families.

**Implications for the Profession and/or Patient Care:**

Progressing migration experience of international nurses can improve retention and augment improving patient care and outcomes.

**Patient or Public Contribution:**

No patient or members of public contributed to this research.

**Summary**

- The study establishes greater insight of international nurse migration experience in the first 2 years post‐employment.
- The study discloses importance of recognising international nurse migration experience from both personal and professional perspectives.
- This research impacts issues of global nurse migration.

## 1 Introduction

Whilst challenging to exactify the data, the 2020 *State of the World's Nursing Report* (SOWN) cite the nursing workforce at 27.9 million, with an estimated shortage of 5.9 million (WHO 2020). The projection is workforce numbers will increase over the next 10 years, however, so will nursing shortfalls, thus countering any gains made (ICN 2021). By way of mitigation the International Council of Nurses (ICN) (2021) advises immediate action to improve nursing retention to lessen turnover and minimise exigence of constant recruitment cycles.

International nurse migration adds to the complexity of nurse shortages (Organisation for Economic Co‐operation and Development (OECD) 2021a). Nearly all nurse shortages are concentrated in low and lower middle‐income countries in the global south, and yet nurses, as highly skilled economic labour migrants, are predominantly moving away from these areas to the more prosperous global north (Buchan, Catton, and Shaffer 2022). Many host countries' healthcare systems are contingent on having a steady inflow of migrant nurses (OECD 2021b) and whilst migration has an important social, economic and political impact with remittance for donor countries recognised by the United Nations (Koser 2016), it is heavily cautioned systems look to being self‐sustainable, because dependency on migration cannot be a single or permanent solution in lieu of training domestic nurses (Buchan, Catton, and Shaffer 2022).

England, in the global north, has in recent years significantly increased filling nurse vacancies through international recruitment. In 2022, numbers of international nurses achieved almost half of the new nurse workforce in the United Kingdom (UK) (Nursing and Midwifery Council [NMC] 2023). This steep upsurge is concerning the UK is now overdependent on overseas recruitment to populate its nursing workforce (Buchan, Catton, and Shaffer 2022). As international migration grows in influence and increasing globalisation summons we now move as one global healthcare system there is a realisation that the nursing workforce is severely depleted worldwide, with insufficient replenishing stocks (Buchan,Catton, and Shaffer 2022; ICN 2021). As such, it is advised each healthcare system independently conducts periodic nursing workforce impactassessments to contribute managing risks to providing healthcare across the globe (Buchan, Catton, and Shaffer 2022; ICN 2021).

Considering international nurse retention is propositioned as the single most effective way to stabilise the healthcare workforce and is essential in times of growing shortages, it is remiss that factors related to retention of international nurses in host countries are underexplored in research (Villamin et al. 2024). Identifying ways to improve retention could advise individual countries taking accountability for supporting international nurses' experiences of migration to improve outcomes. It is advocated that if each healthcare system acts, workforce gains could extend singular borders and collectively improve global sustainability (Buchan, Catton, and Shaffer 2022; ICN 2021).

Turnover is an antecedent to lack of retention and both turnover and turnover intention in nurse migration is increasing, meaning that the threat to nurse retention is growing (ICN 2021; Buchan, Shaffer, and Catton 2018). Definitions of nurse retention, turnover, and turnover intention are criticised as indistinct and it is cautioned that ambiguity can lead to misrepresentative and inconsistent conclusions (Bolt, Winterton, and Cafferkey 2022). It is therefore useful to be clear in definition that retention is workforce stability when nurses stay in the profession or organisation; turnover is the act of leaving an organisation, and turnover intention is the likelihood of leaving an organisation at some point or soon (Villamin et al. 2024; Buchan, Shaffer, and Catton 2018; Oliveira et al. 2018). This suggests that to take effective action to support international nurses to stay, we must first recognise factors affecting retention (Pressley and Garside 2022; Pressley et al. 2023).

Many factors influence the experience of migration (Buchan, Catton, and Shaffer 2022). Migrating to live and work thousands of kilometres away from home and often being separated from partners and families for an undetermined period is a significant life event (Bond 2022). So too is choosing to build a life and career and working in the longer term as a professional in another country; both described as easier when comprehending achieving fulfilling and sustainable personal and professional lives (Buchan, Catton, and Shaffer 2022; Davda, Gallagher, and Radford 2018). Much is written about initial motivations for nurse migration such as opportunities for career development and improved quality of lives (Pressley et al. 2022; Alexis and Shillingford 2015). However, there is limited research about international nurses' experience of life outside of work (Buchan, Catton, and Shaffer 2022; Palmer, Leone, and Appleby 2021). An absolute is that the two exist mutually and are inextricably linked, meaning that it is important to consider both professional and personal issues to fully appreciate the experience of migration and how this will affect international nurse retention (Bond 2022; Mosley and Irvine 2020).

Individual decisions to stay or leave working in a host country are multifaceted and often influenced by matters endogenous to the health systems (Young et al. 2014). There is inadequate knowledge of contemporary nurse migration issues affecting experiences of life outside of work, such as housing and the cost of living, reasons for family separation and navigating challenges of living in trans‐national families, social support networks and community incorporation and how experience of migration impacts mental well‐being (Villamin et al. 2024). For this reason and whilst ever issues are underexplored, we cannot reliably forecast if international nurses will be retained working across international borders (Villamin et al. 2024; Bond 2022; ICN 2021).

With larger numbers of international nurses than ever migrating to England, there is a need to ask pressing questions about contemporary migration and experiences working within the healthcare system and living in England (Bond 2022; ICN 2021). Policies in England in the medium‐to longer‐term have outlined intentions to increase domestic nursing workforce supply and to reduce global nursing workforce burden; that said, presently there are many international nurses working in England and retention must be a priority (The National Health Service [NHS] Long Term Workforce Plan, 2023). Therefore, to gain insights, this study aimed to explore the experiences of internationally educated nurses' first 2 years working and living in England (Pressley et al. 2022; Buchan, Catton, and Shaffer 2022).

## 2 Methods

Research design succeeded a systematic review of international nurse migration identifying further research of personal and professional experiences of living and working in England was needed (Pressley et al. 2022, 2023).

### 2.1 Design

Design is exploratory mixed methods survey. Integrating quantitative and qualitative components offered compounding benefit. Quantitative methods framed context of enquiry and where suitable qualitative open free text boxes captured participants' expression. Quantitative design strengthens the size, representativeness and diversity of the study and open‐ended questions in quantitative surveys result in more respondent‐focused studies and more accurate dynamic data stories and can solicit authentic and unexpected feedback to capture diversity of response or nuance of opinion. Free text boxes were designed into the survey questions as optional, meaning if the respondent should choose to provide a narrative response, what they shared was up to themselves and they could write whatever they wanted in their own words, with no restrictions on the length of answers (Punch 2009). This design planned to meet the aim of the study.

Narrative thematic analysis using a coding system intended to classify completer and more meaningful analysis and conclusions from the data (Dawadi, Shrestha, and Giri 2021; Creswell and Plano Clark 2017). Design incorporated methodological strategies to ensure trustworthiness by following the four principles of Lincoln and Guba (1985). Reporting participant voice through quotations from transcribed text connected data and served to disqualify researcher's biases, motivations, or perspectives (Lincoln and Guba 1985).

### 2.2 Sample/Participants

A sample size calculation was not conducted for this calculation. Unlike a comparative study such as a Randomised Controlled Trial, in which there are ethical disbenefits in under‐ and over‐recruitment, in this study there is no optimal number of participants: the aim was simply to maximise precision and diversity by recruiting as many participants as possible.

Piloting the design with a small number of international nurses provided reassurance the questionnaire is valid and did not highlight any problems with questions (Punch 2009).

### 2.3 Data Collection

The survey design was a 5‐point Likert scale (with options *Strongly disagree*, *Disagree*, *Neither agree or disagree*, *Agree*, *Strongly agree*), closed choice items and open text boxes were applied in the questionnaire. Participation was open to all internationally educated nurses recruited to work in England for less than 2 years. Participants were asked about their professional backgrounds, previous nursing experiences, current job allocation and contractual arrangements and experience of professional integration, communication and perceptions of inclusion and belonging. Considerations of life outside of work that delve into experiences of integration into communities including housing situations, community networks, financial situations, plans and aspirations for the future and the impact of migration on health and well‐being were explored. The study used the short Warwick–Edinburgh Mental Well‐being Scale (S‐WEMWBS) to measure mental well‐being among study participants.

The questionnaire (managed and distributed via Qualtrics software) was open for responses from August 2022 to October 2022. Convenience sampling was adopted. Software was programmed to ensure individual submitted no more than one survey response. Information about the survey and internet link to completing the survey were widely distributed through social media (*X*) and by email from nursing leaders answerable for international nurses employed across NHS regions (North East & Yorkshire, North West, Midlands, East of England, London, South East and South West) of England.

In total, 773 participants submitted valid survey responses.

### 2.4 Data Analysis

Research conducted descriptive and inferential analyses of quantitative data and subjected the open‐ended survey responses to thematic analysis. There was constraint and advantage to this design. Methodologically, literature puts forward that completing open‐ended response surveys requires greater resource of time for participant to complete and researchers to analyse than surveys with closed‐ended questions (Dillman 2007). However, adopting a mixed methods approach and adding open question to the closed questions significantly enhanced research findings and allowed researchers to draw comprehensive reasonable and meaningful data conclusions from greater numbers of participants (Suter 2012).

Descriptive processes provided the initial analysis of the quantitative data, including frequency summaries of individual variables and cross‐tabulations. Associations between of specific factors of interest collected in the survey were analysed using the chi‐squared test for association to assess generalisability of associations to the wider population of international nurses. The direction of any effect was noted and the magnitude of any effect was reported using the phi‐statistic. For the purposes of this analyses, Likert‐type survey items were dichotomised into positive responses (*Strongly agree* or *Agree*) versus a neutral response (*Neither agree or disagree*) and negative responses (*Disagree* or *Strongly disagree*) (Garside et al. 2023).

Associations between categorical predictors and outcomes were assessed for significance. Qualitative data analysis was informed by Braun and Clarke (2006)'s six‐phase inductive thematic review process to identify, analyse and report patterns and themes in the research findings. An initial and open coding process was thus established using NVivo version 12 qualitative data analysis software, to classify the categories of information emerging from the research findings. As coding developed it became clear that overlap was present and codes were collapsed and initial themes identified and compared and synthesised with the quantitative findings (Garside et al. 2023). Participant quotes were used in findings as evidence of the themes and sub‐themes and to provide a richness of insights for the reader.

### 2.5 Ethical Considerations

The Health Research Authority tool established research did not require formal NHS Research Ethics Committee approval. Ethical approval was granted by the University of Huddersfield.

### 2.6 Findings

Quantitative data from the 773 participants was taken and data and corresponding percentages were calculated based on the responses valid for analysis. As a result, the total number of responses considered may vary slightly in different sections.

### 2.7 Demographics

Table 1 presents a summary of respondent demographics.

**TABLE 1:** Sample characteristics.

Demographics	Category	Frequency	Valid percentage (%)
Gender (*n* = 760)	Female	625	82.2
	Male	135	17.7
Country of birth (*n* = 772)	India	321	41.6
	The Philippines	182	23.5
	Nigeria	134	17.4
	Rest of Africa	101	13.1
	Rest of the world	34	4.4
Marital status (*n* = 705)	Married	447	63.4
	Single, Never Married	258	36.6
Family compositions (partner living status (*n* = 454)) Family compositions children (*n* = 381)	Partners live in England	322	70.9
	Partners do not live in England	132	29.1
	Hope for partner to join in future	128	96.9
	Have children under 18	381	49.2
	All children live in England	223	58.5
	Some children live in England	13	3.4
	No children live in England	145	38.1
Plans for family members to join living in England (*children n* = 153) (*family n* = 312)	Hope for children to join in future	153	96.8
	Hope for other family members to join	312	40.3

Asking about past employment revealed, 252 international nurses (32.6%), said England was the second or third country they had worked in after completing training in their countries of origin and for many the choice to onward migrate was based on their family priorities, as reflected in comments:

I chose the United Kingdom because here I can settle with my family.

I was able to get a student visa for my daughter to continue her education in England kind courtesy of my employer.

And (as shown in Table 1), 96.8% of those with children living separate hoped to reunite living together in England and 40.3% living separate from other family members hoped to reunite living together in England.

### 2.8 Professional Integration

Professional integration explores key areas within organisational and workplace domain, these are: recognition of prior professional experiences, induction processes, career development systems and role of line managers in providing support, communication barriers in the workplace and understanding of human resource policies.

Table 2 presents a summary of professional integration items relating to respondent employment.

**TABLE 2:** Quantitative responses to role and organisational items.

Item	Category	Frequency	Percentage (%)
Duration of employment (*n* = 773)	< 1 month to 2 months	100	12.9
	3 months to 5 months	153	19.8
	6 months to 12 months	301	39.0
	1 year to 2 years	219	28.3
Employment band (*n* = 773)	Band 3	71	9.2
	Band 4	85	11.0
	Band 5	603	78.0
	Band 6	13	1.7
	Band 7	1	0.1
Career development conversation with line manager or mentor (*n* = 773)	Yes	314	40.6
	A partial conversation	130	16.8
	No	329	42.6
Ability to communicate with patients (*n* = 768)	Positive response	550	71.6
	Neutral response	124	16.1
	Negative response	94	12.2
Ability to communicate with colleagues (*n* = 773)	Positive response	573	74.1
	Neutral response	94	12.2
	Negative response	106	13.7
Understanding of organisational employment benefits (*n* = 773)	Good understanding	276	35.8
	Fair understanding	396	51.2
	No understanding	101	13.0
Payback requirements for leaving contract (*n* = 773)	Required to payback	528	68.3
	Not required to payback	195	25.3
	Unsure	48	6.3

#### 2.8.1 Recognition of Prior Experience

Most respondents were employed in a band 5 position, or below, with just 1.8% (*n* = 14) of respondent working at band 6 or higher (see Table 2). Comments revealed that many felt that their years of working were overlooked in their band allocation by employers in England:

I have realised that they did not consider my previous experience and those who have 25 years of experience and 5 years of experience falls under the same pay category which is completely wrong…

Others, voiced recognition of experience but stated no additional pay increment in wage:

I have 13 years of experience… when in work they consider my experience but not in salary.

I am a nurse with overall 11 years of clinical experience…it is better if NHS would consider our previous experience for salary package.

#### 2.8.2 Specialist Nursing Experience

Many international nurses migrating to England had specialist nursing skills and experience. Which have benefits for both the employee and employer. Some international nurses narrated:

I used to work in the rehabilitative mental health service back in my country. Working here at the rehabilitation ward made my adaptation and transition into the system faster.

I am very blessed that I'm still in the same allocation as it helped me big time in adjusting…

Respondents expressed greater satisfaction when they had choice of work area, but felt devalued when previous experiences were not recognised:

At times, it is demotivating that a system may accept your qualifications as a nurse but disregards the experience thereof…

I attended the interview as an ED nurse. But got the posting in ward …I am disappointed…

#### 2.8.3 Induction Processes and Supernumerary Periods

Comprehensive and supportive induction processes and supernumerary periods enhance experience of professional integration. Qualitative findings identified a mixed response to satisfaction with induction programmes. Some felt induction programmes were not always comprehensive enough to meet needs, as reflected below:

Knowing fully well that we are not familiar with many of the equipment and procedures, as they are different from what we do back home. Yet, many colleagues expect us to do it right from the first attempt…

International nurses needing to undertake examination to establish registration with the Nursing and Midwifery Council (NMC) UK, described benefiting from practical employer support with preparation for the examination:

They allocated a mentor for me, so that I can approach at any time for queries and clarification… I have no words to explain about her as such a really supportive teacher.

Unfortunately, there were sometimes obstacles accessing support and individuals had to find workarounds:

I've had to manage and survive on my own and by learning from experiences of my fellow international nurses in my ward…

It would be highly beneficial if you put new staff under a senior staff to learn about the routine in our department. Me, I am not from an English‐speaking country and no staff in my ward is from [country of origin] hence, even with 12 years of clinical experience I'm struggling a lot in the ward. There should be clinical instructors in every ward…

#### 2.8.4 Leadership and Management Support

Table 2 shows variation in having career development conversations with line managers. Comments confirm importance of having clear professional development frameworks and leadership. International nurses described the benefits:

I have had a conversation with my line manager about my career. It was helpful and I got to know various opportunities in front of me.

The presence of clinical nurse educators and supportive managers have played a great role in my development as a UK nurse…

My manager encouraged me to apply for the post of Band 6 and enrolled me into university‐based course.

Line managers, clinical educators and/or mentor roles were recognised as effective in bridging personal and professional support offers:

…very supportive manager who actually takes time to sit and listen to me when l am faced with work or even personal issues…[including] … support when I could not understand things, emotional support when I felt homesick and good guidance about how I can improve…

International nurses who had not had opportunity for conversations, explained feeling unvalued to a point of considering leaving England:

There's no opportunity to have such conversations as there is no support. My line manager doesn't even know my name, and has never called me in for a discussion or see how I am fairing…

No one never asked or given opportunities… I am just working without a path or career goals…

#### 2.8.5 Communication and Professional Integration

Supporting international nurses with communication when needed can assist professional integration. Often international nurses described only needing support in the initial period of adjustment:

Getting into a new environment requires learning the accent. But the more I speak and interact with people, the better it becomes … I am just adapting but I guess I will get a grip of it pretty soon …

I am used to hearing the Queen's English, Irish and Scottish from films but I am not familiar with the [country of origin] accent: that's why I had trouble during the first month …

Even though we have been taught English Language since childhood, it is really difficult sometimes to understand because this is not our native language.

Frequently international nurses communicated without a language barrier, however, when communication issues were reported most were in relation to accents, dialects, colloquialisms and slangs:

Being relatively new in England, I am still currently adjusting on how people speak, especially those with accents…and also sometimes I think there are phrases that are new to my ears, I'm not sure if what they meant is figuratively speaking…

International nurses that experienced communication challenges would often ask colleagues to speak audibly, slowly and clearly:

…speak clearly and legibly without rush…

It's a bit difficult to understand the accent of some of them. Initially I had to ask everyone to repeat what they've just said. Now I have and I can feel the difference in that. But I could feel that at least some of them judging and getting irritated …

International nurses described how important clear communication was:

We are dealing with lives, so near misses or error should be avoided.

Quantitative data in Table 2 shows that 71.6% of international nurses reported positively on the ability to communicate with patients and findings show that communication was not raised as a concern for all international nurses:

My first language is English language, and I speak and write better than the Brits in my ward.

I trained with English in my country, so I speak English fluently.

#### 2.8.6 Human Resource Processes and Contractual Payback Clauses

Human resource processes govern professional working arrangements, including issues such as pay, terms and conditions and contracts. There are extensive employee benefits such as sickness and annual leave, human resource procedures and NHS pension schemes working in the NHS. However, in terms of understanding of organisational employment benefits, a minority (35.8%) of international nurses said they had a good understanding, 51.2% had some understanding and 13.0% reported no understanding. This denotes international nurses could be better informed of terms and conditions of employment.

Some international nurses employed in England have contractual clauses written into employment contracts to payback monies rendered in their relocation to England if contracts were left before end dates. International nurses' reflections on this matter were mixed:

Fair enough, since they paid for it for us to come here. As long as it's not more than what they paid for OSCE [examinations to obtain professional UK nursing registration], visa, flight ticket. It's somewhat of a fair deal…

Others conceded requirement for the payback, but advise revision to arrangements, as captured in these comments:

I think it is fair because they paid all the expenses. It's an agreement which I consented to, so I am okay with it. I think they could have reduced the amount of payback. I think anyone who works for a Trust for a year should not be made to pay back anything…

If any party is falling short of upholding the conditions, say if the employer refuses to keep to the terms of the agreement, I feel it's fair to leave without any ‘payback’ in order to allow for equal rights to be exercised.

And some international nurses were very much against this policy as unfair. Disagreement was accentuated in remarks such as:

…We should not be inhibited to leave so long as the reason is valid like if we are not being treated fairly, being intimidated in any forms, or even not being allowed to transfer in a different unit.

### 2.9 Personal Integration and Life Outside of Work

This next section of the findings presents personal integration factors affecting international nurses' life outside of work, exploring housing and accommodation, cost of living, social support and mental well‐being. Many items in tables were expanded through narratives.

Table 3 presents a summary of the responses to housing and living arrangements.

**TABLE 3:** Responses to housing and living.

Item	Category	Frequency	Valid percentage (%)
Number of properties lived in since moving to England (*n* = 730)	1	226	30.9
	2	363	49.7
	3 or more	141	19.3
Housing tenure (*n* = 736)	Private rented	589	80.0
	Employer‐provided	125	16.9
	Social rented	16	2.1
	Owned outright or with a mortgage	6	0.8
Household composition (*n* = 725)	Lived with friends	243	33.5
	Lived with partner and children	237	32.6
	Lived with partner	97	13.3
	Lived alone	148	20.4
Household satisfaction (*n* = 773)	Happy with housing situation	398	51.5
	Neutral about housing situation	179	23.1
	Negative about housing situation	196	25.4
Location of residence (*n* = 773)	Lived in a city or a town	367	47.4
	Lived in a sub‐urban area	194	25.0
	Lived in a semi‐rural or rural area	212	27.4
Support networks in locations of residence (*n* = 771)	Yes	140	18.1
	Partial	197	25.5
	No	434	56.3
Difficulties commuting to work (*n* = 773)	Yes	517	66.8
	Sometimes	107	13.8
	Never	149	19.2

#### 2.9.1 Housing and Accommodation

In the immediate post‐migration period, employers often provide accommodation for a short amount of time. Unanimously, international nurses valued this and many expressed desires for this offer to increase in length of time:

NHS accommodation is really good and safe.

It would be better if the hospital can provide their own accommodation.

Many described constraints having to find accommodation in limited timescales and at high costs and explained having inadequate knowledge of local areas, landlords and guarantor processes:

I did not have enough time to get a place of my own that I really like after the one month given by my Trust elapsed. It was hard getting a new place as I do not know anywhere. I recommend that Trust should give at least 3 months accommodation to enable international nurses to relax first. I had to live in a house sharing toilet with strangers because I do not know where else to go and makes me feel bad.

Often, international nurses described making compromises and sacrifices with housing choices and 66.8% of respondents described difficulties commuting to work, intimating they are living further away from work than they would prefer:

Because of the fact that most landlords and agents refused to rent out the accommodations… I have ended up renting a place that is very far from my work.

Not where I would have loved to live but due to the difficulty in getting an accommodation, guarantor and all, and no employer's support…

And pressures to find housing were compounded when looking to host families:

I live where no children are allowed, so I will need to move out when family joins.

It's enough to accommodate my family and close to the hospital though [it] took me more than 5 months to secure the house.

Constrictions with processes often limited and forced housing decisions:

… they are asking so many documents…

Unable to get a guarantor so [I] have to share.

These examples could explain why in the short term, many international nurses reported turmoil of moving accommodation several times in the early stages of migration, as illustrated in Table 3. Six respondents (0.6%) owned housing outright or with a mortgage, meaning almost all were living in rented accommodation. It is not defined if home ownership was dismissed as untenable, or if this was not wanted.

What also distinctly came through the survey findings were factors that made respondents feel satisfied with housing, such as wanting safe, well‐maintained, suitable accommodation, conveniently located close to shops, work and transportation:

The area where I live is peaceful and quiet. It is also near the town for shopping and places where I can visit which is great.

My neighbourhood has a good environment and is accessible to all facilities such as shops and bus stops which enable me to get to places, particularly to work.

Close to work, not too expensive and comfortable.

Quiet, peaceful and we have our privacy.

Makes me feel safe as well, as it's not a dodgy area.

However, compromises were often made regarding location, property size, privacy and cleanliness of living conditions:

The cost of renting is high, and I had to rent the accommodation that was available, not what I would have preferred.

It is very far from the hospital where I work. I have to spend significant amount of money to travel by bus or if not, I have to walk 45 min back and forth just to ride on the free shuttle. Sharing bathrooms and kitchen with a lot of tenants is also stressful especially with hours of use, taking turns and cleanliness issue.

I didn't find a place to rent near the hospital I work at, so I settled in a distant one not knowing it was not the safest area to live in. Tales of crimes and not good, stuff has happened around the area. Luckily, I have never encountered one. Although, I often see police cars in our street.

There was a mixed picture of satisfaction with living arrangements and housing priorities of international nurses were seemingly dependent on factors most important to individual circumstances: some valued sharing properties:

I have lived here for 2 years: my landlady has been absolutely wonderful, it's quite near my workplace and I have been blessed with a lovely housemate – whom I consider a family now.

I live with my cohort who became my close friends here in England. This is a big help to reduce homesickness.

Others needed suitable accommodation for their children and family:

The house is small for my family. I have 3 teenage children: 1 girl and 2 boys, we live in a two‐bedroom apartment.

My concern is that if I want another kid, then I have to search for 2 bedrooms in order to accommodate new family setting, housing rules. BUT it will be a burden financially …

#### 2.9.2 Cost of Living

Responses suggest concern that cost of living in England means that salary may not adequately cover housing costs, living costs and left only a minority of respondents the ability to save or spend on luxury items (Table 4). For example, when asked if monthly basic salary adequately covers housing costs such as mortgage/rent payments, council tax and utility bills, many expressed dissatisfactions at the proportion of salary spent, stating:

Living pay cheque to pay cheque due to high cost of living.

The affordability is something that has been bothering me and that is prompting my relocation to another area.

The house rent is expensive, and it excludes bills. With the current NHS band 5 salary and tax system of the country, it leaves little at the end for savings and personal needs.

Table 4 presents a summary of cost‐of‐living responses.

**TABLE 4:** Likert responses to cost‐of‐living items.

Item	Category	Frequency	Valid percentage (%)
Monthly salary covers housing costs (*n* = 772)	Positive response	172	22.2
	Neutral response	166	21.5
	Negative response	434	56.2
Monthly salary covers living costs (*n* = 772)	Positive response	173	22.4
	Neutral response	187	24.2
	Negative response	412	53.3
Ability to save or spend on luxury items (*n* = 773)	Positive response	66	8.5
	Neutral response	116	15.1
	Negative response	590	76.3

International nurses related only just managing financially and expressed worries about funding contingencies and emergencies:

I am spending my whole salary for rent, bills and groceries. If any emergency happens, I don't have any money to [buy] flight ticket as well.

Don't know what to do if any emergencies happened.

International nurses described financial reliance on spouses to meet living costs:

I can afford my rent at this time because I and my spouse work.

And there were accounts of international nurses frequently working extra shifts as a routine way of affording everyday living costs:

with the increasing cost of expenses here, my salary is not enough. I am working extra shifts and now even these are not enough.

Qualitative findings describe some international nurses, experiencing an imbalance between sacrifice made migrating to work in England, and financial reward gained:

The Trust needs to remember that we left our homes and lives… It is difficult on this salary.

We do so much as nurses and earn so little. I struggle monthly to ensure my income is enough for my family. If my Trust had considered my level and years of experience and placed me on the last band 5 salary rate like other Trusts do, I wouldn't have had to struggle this much.

And for some, high cost of housing and living may affect long term plans to remain working in England:

The salary is adequate for single and not people with kids. Bear in mind that we have no access to public funds: childcare cost alone could gulp all my salary for the month if I don't strategize. Coupled with other amenities charges and the cost of an apartment if you've got kids. You can't have your kids in a shared apartment of all bills inclusive or in a one‐bedroom flat…it is expensive to have a family as an immigrant. So, to your question, my salary has never been enough since my kids moved to England to join me, but what can I do?

Cost of living was described as a make‐or‐break issue for continuing to live and work in England:

If it comes to a point where my salary can no longer sustain the cost of living, then I will leave.

And some respondents described the potential option to change longer term plans to stay working in England:

I came with a plan to stay lifelong but now I [have] decided to migrate to Australia which pay better salary.

I really find it difficult to stay here lifelong because of the high expenses and less salary. It's difficult to settle with a family in such a situation so I will consider looking for work in a different country.

With my current salary, pressure from work and short staffing, I am now thinking of migrating to other western country with better healthcare system.

#### 2.9.3 Community Incorporation and Social Support Networks

Most international nurses had no (56.3%) or partial (25.5%) support networks in locations of residence and qualitative examples shared how this impacted individuals:

Being far from your loved ones (family and friends) while working in another country with different culture, etc…you cannot help but feel like sometimes, it's hard to penetrate the social barrier caused by the cultural differences.

Connections provided an important source of information exchange, as well as helping to feel supported and accepted:

My neighbours are friendly; we interact like family members. They are amazing people, and they have helped me with many things since I moved here, everyday things which are often difficult to understand as someone who is new here.

Most of the people when I go outside and buy groceries or go to work, we don't speak much but they are very hospitable, very kind and polite. This why I felt accepted here.

International nurses also recounted the importance of socialising outside of work with people of the same nationality or backgrounds as important to incorporation:

I primarily mix with [people from my country of origin], and we cook together and enjoy most of our traditional dishes that we miss eating back home. Doing this strengthens our friendship and support for one another, and I can learn about their experiences here and they can learn about mine too.

#### 2.9.4 Reasons for Family Separation

Adjusting to living and working in different country without family was found to be one of the most difficult factors of migration for international nurses. How this impacted was not explicitly defined; however, some reasons for continued separation include financial constraints, visa processing issues, difficulty obtaining jobs for partners, planned delays and unwillingness of the family to relocate. Insights were shared that:

The rent and expenses are high with low salary and heavy taxes. As such, I cannot save enough for their travel documents.

On my current salary, I cannot afford the accommodation and childcare cost necessary to live with my son in England as a single mother with no other benefits and no family members to assist with childcare.

I need to be financially prepared first before bringing my partner and kids here. My current financial situation will not permit me to take on the extra burden of bringing my family over.

We are still waiting for the approval of the visa of my husband and two children.

Because he is in a banking position back home which pays well and will not get an equally good job here since he didn't School in England.

Assistance with educational support and affordable childcare costs helped reunite families. Such as in these examples shared:

A non‐expensive facility was provided by the NHS to care for my kids while at work. That eased the pressure on me a lot.

I was able to get a student visa for my daughter to continue her education in England, kind courtesy of my employer.

Some international nurses chose to wait until they were familiar with environments and work schedules, or until they had secured nursing qualification in England, before starting reunion plans with families:

I can bring my partner and kids to the UK after the successful completion of my OSCE exam.

Because I still want to settle first and make sure she and my kids will be comfortable if they come here.

### 2.10 Mental Health and Well‐being

The study used the S‐WEMWBS to measure the mental well‐being among 773 international nurses. The mental well‐being of international nurses is a critical aspect of their overall experience. The mean score for international nurses in this study was 23.05, slightly lower than found in the general population, but varied by country of origin (Fat et al. 2017). African and Indian nurses reported the highest well‐being scores, while Filipino nurses and those from other regions had lower scores. No significant correlations were found between the mental well‐being and factors like age, housing satisfaction, or family composition.

Qualitative data revealed additional factors affecting well‐being, such as workload stress, unfair work allocations and experiences of intolerance and disrespect from domestic nurses.

The workload is huge. Very stressful physically and mentally.

At times, I feel the international nurses are expected to work more than the locals… I used to be confident, make decisions and know what to do most of the time, having to ask most of the times was making me feel useless. That at times really got to me and made me sad…

… The nursing I was doing in my home country is slightly different from the way things are done here. You need someone to show you how to do things here, but no one wants to work with you. So, it is hard to incorporate yourself when you feel left out and alone. You just go home, cry about it and feel like going back home or quitting. Some colleagues are excellent. When you are together on shift, you learn a lot. Trouble is when you have the colleagues that sideline you, a 12‐h shift feels like forever to complete…

Social support networks, particularly from people of the same nationality, were found to help with feelings of loneliness and homesickness.

My faith has greatly helped me to cope, as well as my personal support network of family and friends.

Last month, I had terrible anxiety about work. I kept crying before going to work but having my partner and family on video call before going to sleep and when I wake up have helped ease my worries. I think having my support system with me here in England would be good for my mental health.

#### 2.10.1 Intentions to Stay Working as a Nurse in England

When asked how long they hoped to stay in England, the majority of 338 international nurses (58.0%) said they wanted to stay in England for more than 10 years, whilst 86 respondents (11.1%) said they wanted to stay between 6 and 10 years; 185 (24.0%) said they wanted to stay between 3 and 5 years and 53 respondents (6.9%) said they intended to stay less than 1 to 2 years. A finding of importance was those who reported being happy with their decision to migrate to England had significantly higher S‐WEMWBS scores than those who were not happy with their decision.

## 3 Discussion

This study explored the experiences of internationally educated nurses' first 2 years in England to advise on retention in an age of contemporary migration. To recap results, data illustrates that nurses migrate from many countries, with different family dynamics, live in unique circumstances and have distinctively individual motivations for migration. Findings confirm international nurses are a heterogeneous population and identifies they have divergent professional and personal retention priorities.

Capturing experiences of internationally educated nurses' first 2 years in England, identified some initial challenges with language and communication and adapting to differences in nursing routines were often limited to a period of early migration, which over time seemingly absolve. This declares international nurses require support with different things at different stages when migrating to work and live in a host country (Adhikari and Melia 2015; Pressley et al. 2022). Previous research advocates that a nurse's positive experiences of orientation and onboarding contribute to improved retention outcomes (Leone et al. 2020). This study found mixed satisfaction with induction programmes signifying there is opportunity to improve retention by consistently providing a comprehensive onboarding experience for international nurses.

Career advancement and development opportunities have bearing on experience of work and retention (Humphries, Brugha, and McGee 2009; Leone et al. 2020). Global literature describes how international nurses are often underrated in their competence by host employers. Underrating and overlooking international nurses can lead to them being placed in junior positions, resulting in feeling unfulfilled and discouraged (Sands, Ingraham, and Salami 2020; Adhikari and Melia 2015). Many nurses migrating to work in England were highly skilled and experienced (Pressley et al. 2023; Dahl et al. 2017), and nonetheless, almost all were employed at band 5, which is an entry level nurse position in England. This designates international nurses in England could likewise feel disillusioned and frustrated and offers employers solution to mitigation (Sands, Ingraham, and Salami 2020; Adhikari and Melia 2015).

Despite calls for transparent and merit‐based opportunities for international nurse career progression (WHO 2020), disappointingly, just over a half of respondents had partaken in a career development conversation with line managers and leaders, meaning there have been missed opportunities to support career development. Younger nurses, with fewer familial commitments have a higher turnover intention whenfurthering qualifications and career opportunities are limited (Hayes et al. 2012). Career development conversations invite opportunity to identifyclear professional development plans and explore career potential and increasing uptake in conversations could improve retention (Humphries, Brugha, and McGee 2009).

In addition to the multiplexity of issues facilitating the realisation of successful professional integration, factors affecting personal integration and life outside of work are also multidimensional and priorities were also noticeably individual and dependent on personal circumstances. In relation to housing, most respondents lived in private rented houses, across a mix of towns, sub‐urban, semi‐rural or rural. There were significant constraints with the process of finding housing and two‐thirds of international nurses had already moved twice or more. There were examples of compromise and sacrifice with housing selections, such as property location, size, or occupancy. Overall, just over half of respondents described being happy with their household situation, meaning there is opportunity for improvements in this area.

When exploring details of individual circumstances, 96.8% of nurses with children living separate hoped to be reunited and 40.3% of all respondents would like other family members to join them living in England. Migrating without family is one of the most difficult experiences (Dahl et al. 2022) and family separation can affect the well‐being of children, especially when the migrant nurse is female, as is the case for many nurses in this study (Bond 2022). The nurses themselves are said to carry an ‘emotional cost’ and it is recommended more attention be paid to the impact of separation and how sending remittance cannot ever fully compensate for being away from partners, missing out on the everyday lives of children grow up, or taking care of elderly relatives (Bond 2022; Li, Nie, and Li 2014). International nurses facing this challenge can be supported through policies enabling accessing benefits of long‐term immigration and family immigration to promote international nurse retention (Alshareef et al. 2020; Leone et al. 2020).

Financial incentives often drive migration (Alonso‐Garbayo and Maben 2009); however, frequently it is not the only motivator and other countries to England pay equal or better salaries, meaning other motivations such as family priorities, career development, or to improve quality of life may be ultimate deciding factors (Dahl et al. 2022). Evidence tells that once economic improvement has been gained or partially realised, other needs become more important such as repatriation with family, social support, recreation and professional fulfilment (Villamin et al. 2024). Once people can secure a sufficient income, acquire social networks and find a home, they are much more likely to settle in the longer term, meaning satisfaction with quality‐of‐life influence positive retention outcomes (Bond 2022; Alshareef et al. 2020; Koser 2016). This study revealed that international nurses in the first few years of migration were often not satisfied with their economic status, housing, cost of living and social support, submitting explanation why almost a third do not plan to stay living in England for more than the next 5 years.

Another risk to transient migration and reason international nurses may not be retained in the longer term is that England has in recent years openly removed barriers to international nurse recruits (Buchan, Catton, and Shaffer 2022). There is a concern that when countries make gaining nurse employment easier, they may increase the threat of ‘stepwise migration’; which refers to nurses using countries as ‘stepping stones’ until they reach a preferred destination (Villamin et al. 2024; Carlos 2014). Globalisation means that the world is now a more connected and interdependent place and because of technological communications and transportation revolution, nurse migration is at an all‐time high, with increasing numbers of people migrating several times during their lives, often returning home at intervening periods (Bond 2022; Koser 2016). In recent years, typical patterns of migration worldwide have changed and for example circular migration when individuals move back and forth between host and source countries, or temporary migration, are indicated as future migration behavioural norms (Buchan, Catton, and Shaffer 2022; Bond 2022). Close observation of behaviour patterns of migration is required in this contemporary age of migration where international nurses have licence to move freely between global healthcare systems (Moshiri, Mohammadi, and Yarahmadi 2022; Bond 2022; Koser 2016).

Nurses happy with choice to migrate to England had significantly higher mental well‐being scores and nurses happy with migration decision and having good mental well‐being advocate positively for retention (Villamin et al. 2024). Factors contributing to individuals' happiness with decision to migrate is predicated on unique circumstances and motivation for migration and whilst the detail is not available in these studies' findings, it is recommended that future research with an intersectional focus on international nurse migration could greatly benefit retention planning and amplify outcomes.

Exploring up to 2 years living and working In England has provided novel insight to counsel on international nurse retention in an age of contemporary migration. However, this timeframe limits the capture of information about renewal of employment visas and family visas, or events such as revalidation where international nurses are required to make active decisions whether to continue with employment as a registered nurse in England. There is also a concern that confirmation bias may mean that experiences are interpreted by international nurses more favourably for the period of adjusting to living and working in new ways and surroundings and this may need to be factored into consideration with regards to longer term retention intentions (Peters 2022).

## 4 Conclusion

In summary, global deficit of nurses declares it critical healthcare systems comprehend nurse migration to enrich experience and prevent avoidable leavers (Villamin et al. 2024). This study has captured both measurable data and idiosyncratic narrative of international nurse first 2 years working and living in England. Quantitatively, findings identified rudimentary components for securing nurse migration and the essentialness of recognising transferable skills and experience, offering comprehensive and warm welcomes into new organisations and having well‐maintained suitable accommodation in a geographically desirable place that is conveniently located close to shops, work and transportation. Also requiring systems address, is once housing and living costs are deducted from monthly salaries, there must be sufficient financial gains leftover; and international nurses wanting to unite with children and families must be able to do so. Qualitatively, narrative exposed nurse migration as deeply individual, with unique and expansive, professional and personal circumstances, suggesting priorities and what is important and significant is unique and nuanced. Furthermore, with needs transient and priorities changing over the course of migration employment offers must be agile.

This study, albeit accepting its bounded reality of providing a partial worldview in a complex global system, suggests practical ways to improve opportunity for international nurses to experience a reality of living the personal and professional lives they desired when they chose to migrate to England. Conceding also pragmatically, in an age of contemporary migration, when nurses are in high demand and can move freely between healthcare systems, there is no singular unequivocal solution to prevent all avoidable leavers; an overall conclusion suggests England, can only and must, do everything possible to ‘be the preferred destination’. This will allow England's healthcare system to capitalise from benefits of existing international nurse skills and experience, reduce the threat of onward migration to other countries and proactively future‐proof its system as desirable for future recruits as patterns of migration evolve worldwide. This directive determines working towards securing the experience of international nurses through a concordance of congruent policies, organisational culture, leader, line manager and wider network support, as described in this study.

## Ethics Statement

Ethical approval was received from the University of Huddersfield's School Research Ethics and Integrity Committee prior to dissemination of the survey. The research was confirmed by the Health Research Authority tool as not requiring formal NHS Research Ethics Committee approval as it was not medical research or clinical trial and did not involve service users. The survey required informed consent prior to commencing the questionnaire and respondents were assured that confidentiality and anonymity would be maintained and individuals would not be identifiable in any publications from the research.

## Conflicts of Interest

The authors declare no conflicts of interest.

### Peer Review

The peer review history for this article is available at https://www.webofscience.com/api/gateway/wos/peer‐review/10.1111/jan.16543.

## Data Availability

The data that support the findings of this study are available from The University of Huddersfield. Restrictions apply to the availability of these data, which were used under license for this study. Data are available from c.pressley@hud.ac.uk with the permission of The University of Huddersfield.
